# Gartner duct cyst in pregnancy presenting as a prolapsing pelvic mass

**DOI:** 10.2349/biij.3.4.e46

**Published:** 2007-10-01

**Authors:** AV Arumugam, G Kumar, LK Si, A Vijayananthan

**Affiliations:** 1 Department of Biomedical Imaging, Faculty of Medicine, University of Malaya, Kuala Lumpur, Malaysia; 2 Department of Obstetrics and Gynaecology, Faculty of Medicine, University of Malaya, Kuala Lumpur, Malaysia

**Keywords:** Gartners duct cysts, Wolffian duct, magnetic resonance imaging

## Abstract

Gartner duct cysts are the remnants of the Wolffian duct and they are rarely seen in adulthood. We present a case of a pregnant patient with a prolapsing vaginal mass. A diagnosis of Gartner duct cyst was made after MRI was performed. The Gartner duct cyst was drained when the patient went into labour allowing vaginal delivery to be performed.

## CASE REPORT

A 30-year-old primigravida was seen at 27 weeks of pregnancy for a routine check up. Antenatal ultrasound showed a single intra uterine pregnancy and a cystic structure in the pelvis, which was diagnosed as an ovarian cyst in pregnancy. A repeat ultrasound at the authors’ hospital confirmed a single viable foetus in cephalic presentation. There was a cystic mass measuring 7.8 cm x 7.4 cm x 5.9 cm in the left adnexa, which was diagnosed to be an ovarian cyst. During the 36th week of pregnancy, the patient felt a mass descending from her vagina on straining at micturition and defecation.

**Figure 1:**
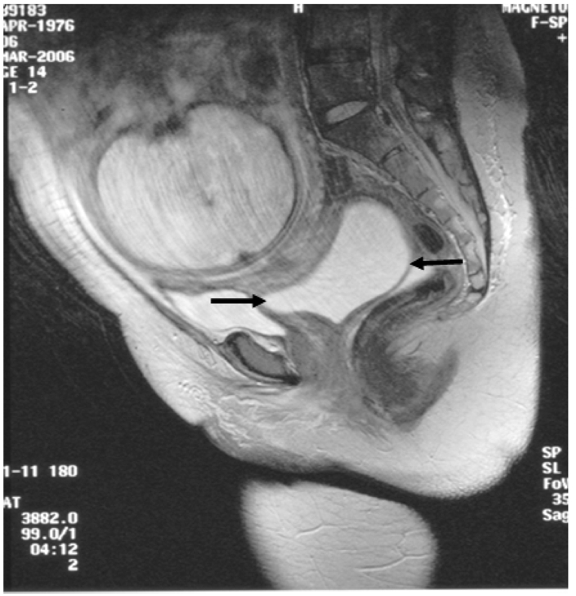
Sagittal T2-weighted MR image of the pelvis shows a cystic mass (arrows) lying posterior to the urinary bladder and inferior to the gravid uterus.

**Figure 2:**
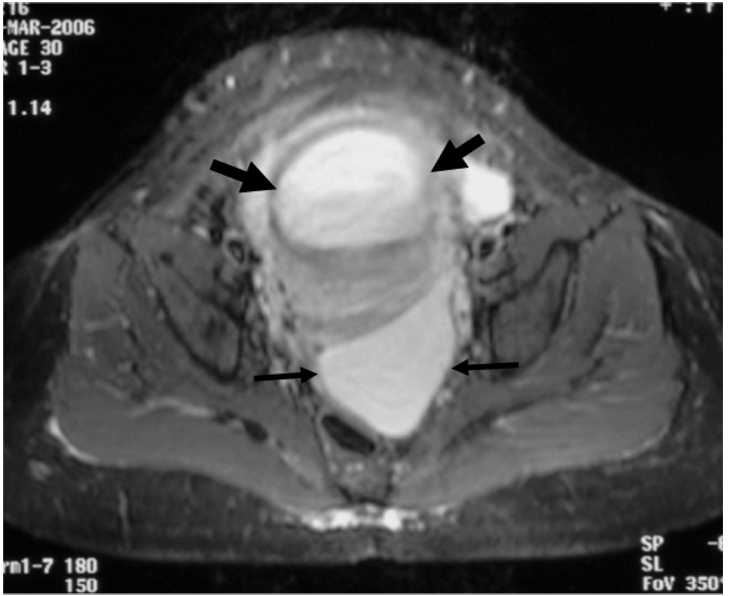
Axial STIR MR image shows a well-defined cystic mass (thin arrows) lying posterior to the gravid uterus (thick arrows).

**Figure 3:**
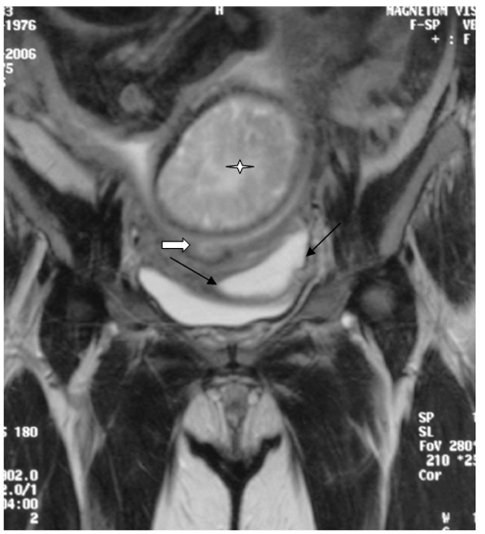
Coronal T2-weighted image shows the cyst (black arrows) lying lateral to the cervical os (block arrow). The fetal brain (asterisk) is seen within the uterus.

A digital and speculum examination of the vagina revealed a cystic swelling measuring 7 cm in size that had a stalk attached to the left vaginal wall, displacing the cervix to the right side. Magnetic resonance imaging (MRI) of the pelvis was performed and revealed a mass in the left lateral vaginal fornix. This mass was homogenous and returned low signal on T1-weighted and high signal on T2- weighted images, consistent with a cyst. The mass extended into the pelvis and measured 8 cm x 3.6 cm x 6.2 cm. The gravid uterus and cervix were displaced superiorly (Figures 1 to 3). No other pelvic masses were seen.

In view of the imaging and clinical findings, a diagnosis of Gartner duct cyst in pregnancy was made.

At 39 weeks of pregnancy, the patient went into labour and under epidural anaesthesia, aspiration of the Gartner duct cyst produced 60 ml of clear fluid. Delivery was then induced. The baby was delivered with vacuum assistance due to poor maternal effort. The cyst fluid was sent for cytology examination where smear microscopy showed degenerating red blood cells, a few lymphocytes and polymorphs with squamous cells. Mother and baby were subsequently discharged well.

## DISCUSSION

The differential diagnoses of a cyst in the lateral aspects of the female genital tract include ovarian cysts, broad ligament cysts, nabothian cysts, Bartholin cysts and Gartner duct cyst.

During the eighth week of embryologic development, the paired Müllerian (paramesonephric) ducts fuse distally and develop into the uterus, cervix and upper vagina, which are lined by a pseudostratified columnar (glandular) epithelium. Wolffian (mesonephric) ducts normally regress in the female, and their remnants include Gartner duct, epoöphoron and paroöphoron. Beginning at week 12 of intrauterine development, a squamous epithelial plate derived from the urogenital sinus begins to grow upward and replace the original pseudostratified columnar epithelium with squamous mucosa [1]. The Gartner duct is the remnant of the vaginal portion of the Wolffian duct. Secretion by persistent glandular epithelium causes cystic dilatation, giving rise to Gartner duct cysts.

Müllerian and Wolffian derivatives can be found at almost any location within the vaginal walls. Clinically, the distinction between Müllerian and Gartner’s duct cysts is of little importance [1].

True Gartner’s duct cysts are usually located along the anterolateral wall of the proximal third of the vagina. In contrast, Bartholin cysts are usually located in the posterolateral wall of the inferior third of the vagina associated with the labia majora [1, 2]. Typically, Gartner’s duct cysts are small and asymptomatic, with an average diameter of 2 cm. When the cysts enlarge they may be mistaken for other structures, such as a cystocele or urethral diverticulum. The largest Gartner duct cyst reported measured 16 cm x 15 cm x 8 cm [4]. The larger cysts can also cause dyspareunia or interfere with normal vaginal delivery [2].

Gartner duct cysts can also be associated with abnormalities of the metanephric urinary system. Although such abnormalities usually present in childhood, awareness of this association should prompt the clinician to image the urinary tract in these patients. Ectopic ureters, besides having direct communications with the vagina and introitus, have been reported to communicate with Gartner duct cysts and cause urinary incontinence [3]. The Gartner duct cyst may also represent an ipsilateral blind vagina, thus lending support to the hypothesis that the distal segment of the Wolffian duct contributes to the formation of the vagina.

MRI is the imaging modality of choice for characterising the cyst [4]. These cysts have been observed in 1%-2% of female pelvic MR imaging examinations [1]. They are typically located in the anterolateral aspect of the proximal third of the vagina. They exhibit low signal intensity on T1-weighted images and high signal intensity on T2-weighted images when they are simple cysts. When there is intracystic protein, mucin, or hemorrhage, they exhibit intermediate to high signal intensity on T1-weighted images. Neither the cyst nor its wall enhances after intravenous contrast injection [4]. Most of these lesions are confined to the vaginal walls, but the larger cysts can extend into the ischiorectal fossa.

Other cysts that can arise in the vagina and cause considerable diagnostic confusion are Bartholin cysts. They arise from the duct system of Bartholin glands. Most cysts involve the main duct only and thus are unilocular. Bartholin cysts are usually unilateral, non tender, tense, palpable masses 1-4 cm in diameter. Most contain sterile fluid and are located in the posterior part of the labia majora. The cysts are often asymptomatic unless they become enlarged or infected, whereby contrast administration demonstrates pericystic enhancement on computed tomography (CT) and MR [4].

Nabothian cysts are retention cysts of the cervical glands. They are caused by chronic inflammation with scarring of the cervix, which leads to occlusion of the lumen of the cervical glands. They appear as single or multiple well-circumscribed cystic lesions in the cervical fibrous stroma and can grow considerably large. Cystic accumulation of mucus within the dilated glands accounts for the MR appearance [4]. Most of them show high signal intensity on T2-weighted images. On T1 -weighted images, most were isointense with urine or muscle [4].

Paratubal (paraovarian) cysts develop within the broad ligament. They arise most commonly from either the mesothelial epithelium of peritoneal inclusions or vestiges of the paramesonephric ducts and rarely from remnants of the mesonephric ducts [2]. Many Gartner duct cysts drain spontaneously or are aspirated, as in this case. If surgical treatment is indicated, marsupialisation or simple transvaginal excision is usually adequate [6].

In conclusion, the differential diagnosis of a cystic lesion found in the lateral aspect of the female genital tract should include Gartner duct cysts. MRI is the imaging modality of choice in confirming the diagnosis.
